# Health effects and externalities of the popularization of sanitary toilets: evidence from Rural China

**DOI:** 10.1186/s12889-023-17192-4

**Published:** 2023-11-11

**Authors:** Yingwen Gu, Wanli Zhou, Tan Zheng, Fang Huang

**Affiliations:** 1 College of Humanities, Hubei University of Chinese Medicine, Wuhan, China; 2 Hubei Shizhen Laboratory, Wuhan, China; 3 School of Finance and Public Administration, Hubei University of Economics, Wuhan, China; 4 Collaborative Innovation Center for Medical Insurance Reform, Hubei University of Economics, Wuhan, China; 5 School of Public Administration, Zhongnan University of Economics and Law, Wuhan, China; 6 Tongji Medical College, The Central Hospital of Wuhan, Huazhong University of Science and Technology, Wuhan, China

**Keywords:** Sanitary toilets, Health effects, Externality, Children, Two-way fixed effects

## Abstract

**Background:**

This study aimed to assess the impact of the increased prevalence of sanitary toilets in rural areas on the health of rural residents, and whether the popularity thereof has a positive externality. This study investigates whether the broader use of sanitary toilets has had a positive effect on the health of people who do not have access to them.

**Methods:**

Data from the China Family Panel Studies from 2012 to 2014 and a two-way fixed effect model were used to investigate the relationship between the prevalence of village sanitary toilets and the health of rural residents of all ages.

**Results:**

The results showed that: (1) the increase in the prevalence of sanitary toilets in villages is conducive to improving the health level of rural residents; (2) the widespread adoption of sanitary toilets in rural areas has improved the health of not only residents with access to these toilets but also residents without access; (3) the health of children is more sensitive to improvements in sanitary conditions of toilets; and (4) there are significant regional differences in the impact of the popularity of sanitary toilets on the health of rural residents.

**Conclusions:**

This study found that the popularity of sanitary toilets has externalities, improving not only the health of residents who use them but also the health of other residents. This study enriches the literature in the field of health effects of sanitation improvement, while providing a reference for developing countries to further enhance the living environment in rural areas. In the future, the popularization of sanitary toilets should be vigorously promoted to reduce the incidence of diseases.

**Supplementary Information:**

The online version contains supplementary material available at 10.1186/s12889-023-17192-4.

## Background

The toilet, a typical representation of environmental sanitation facilities, is an important indicator of a civilized society [1]. The sanitary level of the toilet is closely related to residents’ health. Excrement pollutes food and water sources, causing intestinal diseases that kill 1.5 million children under the age of 5 every year, which is higher than the death toll caused by AIDS and malaria [2]. COVID-19, which has wreaked havoc worldwide, has also been identified as posing a risk of fecal-oral transmission [3]. According to data from the World Health Organization, as of 2015, one-third of the world’s population still used unimproved sanitation facilities, and 946 million people defecated in the open and lived in poor rural areas [4].

Unsafe management of human excrement is a major public health risk, especially in low- and middle-income countries [5]. Studies have shown that poor sanitary conditions in toilets can damage health, leading to an increase in the incidence of infectious diseases, and cause environmental deterioration. Toilets are important vectors of disease transmission, and human feces are one of the main transmission routes of many diseases [6]. Pathogens, such as bacteria in feces, can be transmitted through sewage, feces, and human-to-human contact; polluted air can also enter the respiratory system to cause diseases [7, 8].

Adverse effects on health are multifaceted. On the one hand, poor water supply and sanitation conditions will cause intestinal diseases, the most common of which is diarrhea [8]; on the other hand, from a long-term perspective, open defecation may have a chronic impact on health through the spread of bacteria in contaminated soil, affecting human development and cognitive ability [9]. This impact is more serious for women and children [10, 11].

In areas with poor sanitary conditions, extensive open defecation poses a serious threat to the health and development of children [11]. According to data from 2005 to 2010 on the height of Cambodian children, height is closely related to open defecation [12]. Many researchers have found that the average height of Indian children is generally abnormally short, which is closely related to low sanitary toilet coverage [13]. Intestinal diseases, such as diarrhea or dysentery caused by fecal pathogens, reduce the absorption of nutrients, lead to malnutrition in children, cause slow growth in children, and ultimately affect their height [5, 14–18]. The coverage and utilization rates of sanitary toilets will not only affect children’s height development in the short term but also the development of human capital in the long term [19–21].

The popularization of sanitary toilets mainly prevents the spread of diseases, especially via the fecal-oral route, by providing a clean environment to protect human health [22]. Many studies have suggested that improving sanitary facilities can reduce the incidence of related diseases [10]. For example, toilet improvement can benefit health, the environment, society, and the economy, and significantly reduce the incidence of diseases caused by fecal transmission, such as ascaris, infectious diarrhea, and dysentery [23].

Some scholars have studied the externalities of sanitary toilet popularity [24]. Based on their research on Cambodian children’s height, Vyas et al. [13] concluded that the health behavior of neighbors, rather than the health behavior of the family itself, has a greater impact on children’s height. Defecation has significant direct and indirect impacts on children’s health. The indirect impact is mainly due to the externalities of sanitary toilets and fixed-point defecation [12, 25]. Compared with children living in villages without sanitary toilets, children living in villages with sanitary toilets had a 47% reduced incidence rate of diarrhea; one-fourth of this reduction was attributed to direct impacts, while the balance was attributed to external effects.

Based on the existing literature, considerable research has been conducted on the relationship between toilet sanitation facilities and health, but some shortcomings and deficiencies remain. First, the current research mainly discusses the relationship between toilet sanitation facilities and health (primarily focused on diarrhea and children’s height), and predominantly uses data from other developing countries, while little attention is paid to data from China. Therefore, whether the existing research results can be extrapolated to the conditions in China remains to be verified. Second, current research on the externalities generated by the improvement in sanitary conditions of toilets, especially evidence from China, is still relatively lacking.

According to data from China’s Third Agricultural Census, by the end of 2016, only 36.2% of rural households had flush toilets, meaning that most still used dry toilets [26]. Poor toilet hygiene causes approximately 17 million Chinese households to suffer from serious health problems every year [27]. The Millennium Development Goals recognized that eliminating open defecation is essential for improving the health, nutrition, and productivity of the population in developing countries. For countries such as China and India, whether and to what degree investment in sanitary toilets and other facilities will bring about health improvement has become a matter of concern.

Therefore, this study used data from the China Family Panel Studies (CFPS) and the two-way fixed effect model to evaluate the health benefits and externalities generated by the increased prevalence of sanitary toilets in rural areas of China. This study can provide a reference to further improve the toilet renovation plan in rural areas and continue to promote public health development in rural areas. It can also provide an empirical reference for the promotion of sanitary toilets in other developing countries with relatively inadequate toilet sanitation facilities.

## Methods

### Data

The data used in this study were derived from the CFPS, which is a national, comprehensive social survey project. The Social Science Survey Center of Peking University is responsible for questionnaire implementation and data cleaning. The CFPS conducted its baseline survey in 2010, and subsequently conducted five rounds of full sample tracking surveys in 2012, 2014, 2016, 2018, and 2020. The sample represents 95% of China’s population and covers 25 provinces, cities, and autonomous regions in China. The questionnaire contents include population, economy, education, family dynamics, physical and mental health, etc. It is divided into three levels: individuals, families, and villages (communities). The data derived from the survey can meet the needs of this study for indicators such as the prevalence of sanitary toilets and the health status of family members.

Since 2016, the dataset no longer provides information about domestic toilet types, and this study uses survey data from 2012 to 2014. To compare the impact of the popularization of sanitary toilets on the health of adults and children, we divided the sample into adult and child samples. In this study, the child sample comprises children aged 16 and under, whereas the adult sample comprises the population aged over 16. After data screening, totals of 39,671 adult respondents and 7,263 child respondents were interviewed simultaneously in 2012 and 2014.

### Variables

#### Dependent variables

This study selected self-rated health at the time of the survey (2012 and 2014), discomfort in the past two weeks, and diarrhea in the past two weeks as indicators to measure adult health, and parents’ evaluation of children’s health, whether they were ill in the past month, and the number of times they were ill in the past month as indicators to measure children’s health. The questionnaire items regarding adults’ self-rated health specifically asked “What do you think of your health status?” with possible responses of “5 = excellent,” “4 = very good,” “3 = good,” “2 = fair,” and “1 = poor.” In this study, “excellent,” “very good,” and “good” are defined as healthy, with a value of 1, and “fair” and “poor” are defined as unhealthy, with a value of 0.

Discomfort in the past two weeks and diarrhea in the past two weeks were both binary variables, with 1 indicating yes and 0 indicating no. Young children’s self-rated health was assessed by their parents. The value range is 1–7, where 1 represents the worst health condition, and 7 represents the best health condition. Being sick in the past month is a binary variable: 1 represents sick, and 0 represents not sick. If the children were sick in the past month, their parents reported the specific number of times.

#### Explanatory variables

The core explanatory variable of this study is the prevalence of sanitary toilets in villages; that is, the proportion of the total number of toilets that meet sanitary standards to the total number of households in the village^1^. We referred to the research of Mangyo [28] and Lamichhane and Mangyo [29] and used the question of “whether a sanitary toilet is used at home” to calculate the total number of households in the same village (community) that used independent sanitary toilets as the numerator, and we used the total number of households in the same village as the denominator to calculate the prevalence rate of village sanitary toilets.

#### Control variables

The control variables in this study covered three aspects: (1) personal characteristics, including age, marital status, and education level; (2) family characteristics, including family per capita income, family size, and family living congestion; and (3) village characteristics, including the type of cooking water and whether there was a public garbage facility and service. For the adult sample, we used age, marital status, education level, the number of family members, per capita income of families, household housing congestion last year, cooking water, and whether public garbage is available as control variables. In addition to other variables, the children’s sample also included the variable of whether the child is enrolled in school. The descriptions, types, and possible values of variables are shown in Table 1.

**Table 1:** Variable details

Variable	Type	Values
Self-rated health (Adult)	Dummy	0 = Unhealthy ; 1 = Healthy
Self-rated health (Children)	Continuous	Value range: 1–7
Discomfort in the past two weeks	Dummy	0 = No; 1 = Yes
Sickness in the past month	Dummy	0 = No; 1 = Yes
Number of episodes of illnesses in the past month	Continuous	
Popularization rate of sanitary toilets	Continuous	
Sanitary toilet available for use at home	Dummy	0 = No; 1 = Yes
Age	Continuous	
Gender	Dummy	0 = Female; 1 = Male
Gender of the child	Dummy	0 = Girl; 1 = Boy
Marital status	Dummy	0 = Divorced/Widowed/ Unmarried;1 = Married
Education level	Categorical	1 = Uneducated; 2 = Primary school; 3 = Junior high school; 4 = High school, university, and above
Enrolled in school	Dummy	0 = No; 1 = Yes
Number of episodes of illnesses in the past month	Continuous	
Number of family members	Continuous	
Household per capita income last year (natural logarithm)	Continuous	
Congestion degree of family residence	Continuous	Value range: 1–7
Type of cooking water	Dummy	0 = Other water sources; 1 = Tap water and purified water
Availability of public garbage cans in village	Dummy	0 = No; 1 = Yes

### Statistical model

This study focused on the impact of the popularity of sanitary toilets on the health of rural residents and the external benefits of that popularity. Considering the use of the ordinary least squares estimation method, it is possible to rule out unobservable or easily missed differences between individuals, which may be related to explanatory variables, leading to estimation bias. In this study, a two-way fixed effect model (TW-FE) was selected. The TW-FE model can simultaneously solve the problem of omitted variables that do not change with time but change with individuals, and those that do not change with individuals but change with time.

The results of the Hausman test show that the fixed effects model is better than the random effects model, and the joint significance test shows that the TW-FE model is better than the individual fixed effects model. Therefore, this study used a TW-FE model to investigate the impact of the prevalence of sanitary toilets on the health of rural residents. The model was set as follows:

1 $${Health}_{it}={\beta }_{1}+{\beta }_{2}{ST}_{it}+{\beta }_{3}{Z}_{it}+{\mu }_{i}+{T}_{t}{+\epsilon }_{it}$$

${Health}_{it}$ refers to self-rated health, discomfort in the past two weeks, diarrhea in the past two weeks, parents’ health evaluation of children, whether children were sick, and the number of times illness occurred in the past month. ${ST}_{it}$ represents the prevalence of sanitary toilets, ${Z}_{it}$ represents the control variables, ${\mu }_{i}$ refers to the fixed effect of provinces that do not change with time, ${T}_{t}$ refers to the fixed effect of time, which captures the influence of unobserved variables that vary over time but not across provinces on the dependent variables, and ${\epsilon }_{it}$ represents the random interference term that changes with time. ${\beta }_{1}$ represents the constant term, and ${\beta }_{2}$ and ${\beta }_{3}$ are the coefficients of the explanatory and control variables, respectively.

We used this model, first, to evaluate the impact of the popularization of sanitary toilets in villages on residents’ health (regardless of whether sanitary toilets are used at home). Second, we separately evaluated the impact of sanitation toilet popularization on the health of residents who use sanitation toilets at home and those who do not use sanitation toilets. Finally, we examined whether there are regional and gender differences reflected in the impact of the popularization of sanitary toilets on the health of residents who do not use them at home.

## Results

Tables 2 and 3 present basic information of the adult sample and child sample, respectively. The average age of the adult sample is 45.4 years old, and the average age of the children’s sample is 9.12 years old. The overall self-rated health level of both samples is good, with a positive rating of 65.7% for adults and a self-rated health score of 5.65 for children (range of 1–7). Discomfort experienced during the prior two weeks was reported by 31.5% of adults, of which 2.6% reported diarrhea. By contrast, 23.4% of children fell ill in the prior two weeks, with an average of 1.54 episodes.

The proportion of individuals using sanitary toilets at home is higher among the children sample (40.9%) than the adult sample (27.2%). The popularization rate of village sanitary toilets is also higher for children (41%) than for adults (28%).

**Table 2:** Descriptive statistics of variables (Adult sample n = 39,671)

Variables	Mean (%)	SD	Max	Min	Median
**Self-rated health**		0.475	0	1	1
Healthy	65.7%				
Unhealthy	34.3%				
**Discomfort in the past two weeks**		0.465	0	1	0
Yes	31.5%				
No	68.5%				
**Diarrhea in the past two weeks**		0.160	0	1	0
Yes	2.6%				
No	97.4%				
**Popularization rate of sanitary toilets**	0.28	0.333	0	1	0.12
**Whether sanitary toilet is used at home**		0.445	0	1	0
Yes	27.2%				
No	72.8%				
**Age**	45.4	16.5	16	101	45
**Gender**		0.5	0	1	1
Male	50.48%				
Female	49.52%				
**Marital status**		0.399	0	1	1
Married	80.2%				
Divorced/Widowed/Unmarried	19.8%				
**Education level**					
Uneducated	29.8%	0.458	0	1	0
Primary school	24.3%	0.429	0	1	0
Junior high school	30.7%	0.462	0	1	0
High school	11.4%	0.317	0	1	0
University and above	3.8%	0.190	0	1	0
**Number of family members**	4.60	1.956	1	17	4
**Household per capita income last year (natural logarithm)**	8.70	1.237	0.511	13.688	8.934
**Congestion degree of family residence**	4.37	1.474	1	7	4
**Type of cooking water**		0.499	0	1	1
Tap water and purified water	52.5%				
Other water sources	47.5%				
**Availability of public garbage cans in village**		0.480	0	1	0
Yes	36.0%				
No	64.0%				

Note: The variables in this table are not standardized. To eliminate the impact of price factors or inflation, this study used the per capita household income in 2012 as the base period and deflated the per capita household income according to the consumer price index. To reduce the impact of outliers, this study applied the natural logarithm of per capita household income

**Table 3:** Descriptive statistics of variables (child sample n = 7363)

Variables	Mean (%)	SD	Max	Min	Median
**Self-rated health**	5.65	1.062	1	7	6
**Were you sick in the past month?**	0.291	0.454	1	0	0
Yes	23.4%				
No	76.6%				
**Number of episodes of illnesses in the past month**	1.54	1.064	1	15	1
**Popularization rate of sanitary toilets**	41%	0.398	0	1	0.20
**Whether sanitary toilet is used at home**		0.492	0	1	0
Yes	40.9%				
No	59.1%				
**Age**	9.12	4.327	0	16	8
**Gender of the child**		0.494	0	1	0
Boy	42.5%				
Girl	57.5%				
**Enrolled in school**		0.457	1	0	1
Yes	59.4%				
No	40.6%				
**The number of family members**	5.34	1.935	2	17	5
**Household per capita income last year (natural logarithm)**	8.74	1.215	0.981	13.61	8.873
**Congestion degree of family residence**	4.36	1.486	1	7	4
**The type of cooking water**		0.489	0	1	1
Tap water and purified water	60.5%				
Other water sources	39.5%				
**Availability of public garbage cans in village**		0.50	0	1	0
Yes	48.3%				
No	51.7%				

### Impact of the popularization rate of sanitary toilets on the health of adult rural residents

Table 4 reports the estimated results of the impact of the prevalence of sanitary toilets in villages on the health of adults over 16 years old. Among the results, there is no significant correlation between the prevalence rate of sanitary toilets and self-rated health, but there is a significant negative correlation between the prevalence rate and discomfort in the past two weeks, and diarrhea in the past two weeks, both of which are significant at the 1% level. For each percentage point increase in the prevalence of sanitary toilets, the probability of discomfort in the past two weeks and that of diarrhea in the past two weeks was reduced by 9% (p < 0.01) and 13.4% (p < 0.01), respectively.

**Table 4:** Impact of sanitary toilets’ popularization rate on the health of adult rural residents

	(1)	(2)	(3)
Variable	Self-rated health	Discomfort in the past two weeks	Diarrhea in the past two weeks
Popularization rate of sanitary toilets	0.020	-0.090***	-0.134***
	(0.021)	(0.028)	(0.027)
_cons	-0.212**	-0.237**	-0.066
	(0.101)	(0.120)	(0.122)
Obs.	39,671	35,499	11,200
R-squared	0.127	0.123	0.116

Note: The table presents the coefficients from estimation of the TW-FE model with robust standard errors in parentheses. *** p ≤ 0.01, ** p ≤ 0.05, * p ≤ 0.1

The first three columns of Table 5 report the estimated results of the impact of the prevalence of sanitary toilets in villages on the health of family members over 16 years of age who have these toilets in their homes. The results showed no significant correlation between the prevalence of sanitary toilets and self-rated health, but there was a positive correlation between the prevalence of sanitary toilets and discomfort in the past two weeks and diarrhea during the same period, which was significant at the 5% and 1% levels, respectively.

**Table 5:** Impact of sanitary toilets’ popularization rate on the health of adult rural residents

	(1)	(2)	(3)	(4)	(5)	(6)
Variable	Self-rated health	Discomfort in the past two weeks	Diarrhea in the past two weeks	Self-rated health	Discomfort in the past two weeks	Diarrhea in the past two weeks
Popularization rate of sanitary toilets	0.074	-0.129**	-0.165***	0.082**	-0.085*	-0.132***
	(0.047)	(0.064)	(0.055)	(0.035)	(0.048)	(0.044)
_cons	-0.199	0.436	-0.235	-0.308**	-0.399***	-0.085
	(0.242)	(0.284)	(0.272)	(0.123)	(0.147)	(0.146)
Obs.	10,793	9788	2911	28,878	25,771	8289
R-squared	0.109	0.104	0.125	0.108	0.104	0.118

Note: The table presents the coefficients from estimation of the TW-FE model with robust standard errors in parentheses. *** p ≤ 0.01, ** p ≤ 0.05, * p ≤ 0.1

The last three columns of Table 5 report the estimated results of the impact of the prevalence of sanitary toilets in villages on the health of residents aged 16 years and above who do not use sanitary toilets at home. Among the results, there was a significant positive correlation between sanitary toilets and self-rated health and a significant negative correlation between sanitary toilets and discomfort in the past two weeks and diarrhea during the same period. For each percentage point increase in the prevalence of sanitary toilets, the probability of self-rated health increased by 8.2% (p < 0.05), the probability of discomfort in the past two weeks decreased by 8.5% (p < 0.1), and the probability of diarrhea in the previous two weeks decreased by 13.2% (p < 0.01).

### Impact of sanitary toilet prevalence on rural children’s health

Columns (1)–(3) of Table 6 report the estimated results of the impact of the prevalence of sanitary toilets in villages on the health of family members under 16 years of age. The prevalence of sanitary toilets has significant positive and negative relationships with self-assessed health, sickness in the past month, and the quantity of illnesses in the past month, at the 10%, 5%, and 1% levels, respectively. The results in Columns (4)–(6) show a significant positive correlation between the prevalence of sanitary toilets and the health of family members under the age of 16 who have sanitary toilets at home, both at the 5% level.

**Table 6:** Impact of sanitary toilet prevalence on rural children’s health

	(1)	(2)	(3)	(4)	(5)	(6)
Variable	Self-rated health	Were you sick in the past month?	Number of episodes of illnesses in the past month	Self-rated health	Were you sick in the past month?	Number of episodes of illnesses in the past month
Popularization rate of sanitary toilets	0.499*	-0527**	-1.244***	0.179	-0.673**	-2.360**
	(0.296)	(0.263)	(0.235)	(0.276)	(0.291)	(1.155)
_cons	3.408***	-2.914***	10.467***	3.404***	-2.894***	11.408***
	(0.497)	(0.437)	(1.052)	(0.937)	(0.793)	(1.866)
Obs.	7363	7363	7363	3163	3136	3136
R-squared	0.234	0.604	0.604	0.238	0.633	0.635

Note: The table presents coefficients from estimation of the TW-FE model with robust standard errors in parentheses. *** p ≤ 0.01, ** p ≤ 0.05, * p ≤ 0.1

Columns (2) and (3) of Table 7 indicate that with the increased prevalence of sanitary toilets in villages, the probability and frequency of illness of children in families without sanitary toilets decreases. Every 1% increase in the prevalence of sanitary toilets was associated with a reduction in the probability of illness in children by 52.6% (p < 0.05) in the past month, and the number of illnesses in the previous month decreased by approximately two times that amount (p < 0.01). Therefore, the popularity of sanitary toilets has a significantly positive externality.

**Table 7:** Impact of sanitary toilet prevalence on rural children’s health

	(1)	(2)	(3)
Variable	Self-rated health	Were you sick in the past month?	Number of episodes of illnesses in the past month
Popularization rate of sanitary toilets	0.465	-0.526**	-1.940***
	(0.313)	(0.229)	(0.514)
Obs.	4200	4200	4200
R-squared	0.238	0.588	0.587

Note: The table presents coefficients from estimation of the TW-FE model with robust standard errors in parentheses. *** p ≤ 0.01, ** p ≤ 0.05, * p ≤ 0.1

### Regional heterogeneity analysis

To further investigate the externality of the popularity of sanitary toilets, this study divided adult participants and child participants without sanitary toilets at home, into four groups according to region and gender. We then conducted a heterogeneity analysis. Columns (1)–(3) of Table 8 report the estimated results of the impact of the prevalence of sanitary toilets in villages in the eastern region on the health of adult rural residents, while Columns (4)–(6) and (7)–(9) report the estimated results for the central and western regions, respectively.

In the eastern region, the prevalence of sanitary toilets had a significant negative correlation with discomfort in the past two weeks (p < 0.05); in the central region, the prevalence of sanitary toilets had a significant negative correlation with discomfort and diarrhea in the past two weeks (p < 0.05); and in the western region, the prevalence of sanitary toilets had a significant positive and significant negative correlation with self-rated health and diarrhea in the past two weeks, respectively (p < 0.01; p < 0.05).

**Table 8:** Impact of the prevalence of sanitary toilets on adult rural residents’ health: by region

	(1)	(2)	(3)	(4)	(5)	(6)	(7)	(8)	(9)
	Eastern regions			Central regions			Western regions		
Variable	Self-rated health	Discomfort in the past two weeks	Diarrhea in the past two weeks	Self-rated health	Discomfort in the past two weeks	Diarrhea in the past two weeks	Self-rated health	Discomfort in the past two weeks	Diarrhea in the past two weeks
Popularization rate of sanitary toilets	0.086	-0.203**	-0.131	0.005	-0.193**	-0.155**	0.157***	0.123	-0.129**
	(0.064)	(0.085)	(0.099)	(0.060)	(0.076)	(0.077)	(0.061)	(0.091)	(0.054)
_cons	-0.323	-0.343	-0.129	-0.451**	-0.303	-0.260	-0.241	-0.453*	0.098
	(0.222)	(0.265)	(0.367)	(0.220)	(0.252)	(0.246)	(0.201)	(0.249)	(0.173)
Obs.	9082	8488	4630	19,717	21,935	3430	10,782	9248	3140
R-squared	0.119	0.116	0.139	0.110	0.114	0.118	0.112	0.119	0.160

Note: The table presents coefficients from estimation of the TW-FE model with robust standard errors in parentheses. *** p ≤ 0.01, ** p ≤ 0.05, * p ≤ 0.1

The results in Table 9 show that the prevalence of sanitary toilets has regional differences in their impact on the health of underage rural residents without sanitary toilets at home. Compared with the eastern region, the impact on the central and western regions was greater. With the increase in the prevalence of sanitary toilets, the probability and frequency of illness of children in the central and western regions decreased significantly.

**Table 9:** Impact of sanitary toilet prevalence on children’s health: by region

	(1)	(2)	(3)	(4)	(5)	(6)	(7)	(8)	(9)
	Eastern regions			Central regions			Western regions		
Variable	Self-rated health	Were you sick in the past month?	Number of times ill in the past month	Self-rated health	Were you sick in the past month?	Number of times ill in the past month	Self-rated health	Were you sick in the past month?	Number of times ill in the past month
Popularization rate of sanitary toilets	0.314	-0.819*	-0.031	2.486***	-0.976**	3.459***	0.380	-0.859**	-2.289**
	(1.275)	(0.479)	(1.067)	(0.884)	(0.405)	(1.260)	(1.116)	(0.425)	(1.113)
_cons	2.127	-4.331***	13.727***	3.138***	-1.425	5.626**	3.119***	-3.083***	11.716***
	(2.221)	(1.538)	(3.615)	(1.019)	(1.032)	(2.577)	(1.115)	(1.023)	(2.508)
Obs.	3132	2853	3151	2436	2597	2562	1795	1913	1650
R-squared	0.149	0.563	0.564	0.100	0.602	0.594	0.043	0.612	0.615

Note: The table presents coefficients from estimation of the TW-FE model with robust standard errors in parentheses. *** p ≤ 0.01, ** p ≤ 0.05, * p ≤ 0.1

### Gender heterogeneity analysis

The first three columns of Table 10 and the first three columns of Table 11 report the estimated results of the impact of the prevalence of sanitary toilets in villages on the health of adult men and children who do not have sanitary toilets at home, respectively. The last three columns of Tables 9 and 10 report the estimated results of the health of adult women and girls, respectively. With the increase in the prevalence of sanitary toilets, the health levels of adult men and women have improved, but the health levels of children have not changed significantly. There was no significant gender difference in the externalities of health effects owing to the increase in the prevalence of sanitary toilets.

**Table 10:** Impact of the prevalence of sanitary toilets on adult rural residents’ health: by gender

	(1)	(2)	(3)	(4)	(5)	(6)
	Adult males			Adult females		
Variable	Self-rated health	Discomfort in the past two weeks	Diarrhea in the past two weeks	Self-rated health	Discomfort in the past two weeks	Diarrhea in the past two weeks
Popularization rate of sanitary toilets	0.094**	-0.119	-0.147**	-0.067	-0.158**	-0.231***
	(0.047)	(0.066)	(0.073)	(0.053)	(0.070)	(0.054)
_cons	0.023	-0.218	-0.011	-0.690***	-0.560***	-0.120
	(0.170)	(0.205)	(0.260)	(0.178)	(0.210)	(0.175)
Obs.	20,434	19,734	5764	19,237	19,937	5436
R-squared	0.108	0.103	0.115	0.113	0.108	0.130

Note: The table presents coefficients from estimation of the TW-FE model with robust standard errors in parentheses. *** p ≤ 0.01, ** p ≤ 0.05, * p ≤ 0.1

**Table 11:** Impact of sanitary toilet prevalence on children’s health: by gender

	(1)	(2)	(3)	(4)	(5)	(6)
	Boys			Girls		
Variable	Self-rated health	Were you sick in the past month?	Number of times ill in the past month	Self-rated health	Were you sick in the past month?	Number of times ill in the past month
Popularization rate of sanitary toilets	-2.688	0.111	-0.066	1.252**	-0.021	-0.403
	(2.985)	(1.740)	(4.359)	(0.534)	(0.811)	(0.762)
_cons	-9.094	-8.711**	19.340**	3.371***	-3.259***	0.833
	(6.219)	(3.442)	(8.620)	(0.592)	(0.906)	(0.993)
Obs.	3839	3806	3806	3524	3637	3627
R-squared	0.232	0.716	0.725	0.119	0.596	0.182

Note: The table presents coefficients from estimation of the TW-FE model with robust standard errors in parentheses. *** p ≤ 0.01, ** p ≤ 0.05, * p ≤ 0.1

## Discussion

The estimation results show that with the increase in prevalence of sanitary toilets in villages, especially in rural areas, the health indicators of rural residents have improved (discomfort in the past two weeks, diarrhea in the past two weeks). These findings may be related to improvement in the sanitary conditions of toilets [25], which can effectively prevent or reduce excreta pollution reaching food and water sources, thus reducing the incidence rate of intestinal infectious diseases [24, 30]. This is consistent with the conclusions of other scholars [26].

By contrast, some studies suggest that the main mechanism for sanitary toilets reducing disease incidences is not the blocking of water source pollution [27]. For example, if households lack independent sanitary toilets, exposed human and animal feces (fluids) provide a favorable environment for the breeding of bacteria and viruses, which can cause infections through direct contact or diseases (such as schistosomiasis and malaria) transmitted through mosquitoes and flies [1]. Having a sanitary toilet at home can prevent long-term accumulation and exposure of feces, block the transmission of infectious diseases, and thus improve health [27].

Second, the health effects of the popularization of rural sanitary toilets have strong externalities. This conclusion confirms that improving toilets is beneficial for the entire community. By investigating the impact of their popularity on the health of family members without sanitary toilets, this study confirms that an improvement in their popularity can bring strong health externalities, especially for children [31]. The health effects brought about by the improvement of sanitary conditions in rural toilets have a strong spillover effect – a positive externality closely related to the prevalence of sanitary toilets in the village [32]. There must be enough households using sanitary toilets in order to benefit those who do not use them [1, 32]. One study found that the coverage rate of sanitary toilets in villages needs to reach around 30% for this positive externality to manifest [32].

Third, compared with adults, the popularization of sanitary toilets is more conducive to improving the health of children. Modern medical research shows that in many developing countries and regions, including China, toilet stool (liquid) pollution is responsible for infectious diseases among minors [33]. Compared with adults, the underage population is at a developmental stage of growth, and the poor sanitary conditions of toilets are more likely to lead to diseases related to the immune and digestive systems [34]. Another key consideration is that children who are currently in school can receive more hygiene knowledge and education than adults, which is beneficial for changing their hygiene habits and behaviors [35].

Fourth, regional differences exist in the impact of the popularity of sanitary toilets on the health of rural residents. This result is consistent with previous studies [36], and may be attributable to the high level of economic development in the rural areas of eastern China, the high prevalence of sanitary toilets, and the restriction of the law of diminishing marginal benefits. The higher the prevalence rate, the lower the marginal benefits, and the less significant the health benefits [36]. In the central and western regions, where the prevalence of sanitary toilets is relatively low, the effect of sanitary toilets on health is still at a relatively significant stage. Therefore, the health effects of toilet improvement have been more significant in these regions [36].

Fifth, this study did not find a significant gender difference in the health of children owing to the increase in the prevalence of sanitary toilets. However, some studies have found that, owing to the gender difference between girls and boys, girls are more likely to suffer from diseases related to toilet sanitation, and therefore the positive effect of sanitary toilets on girls is more significant [37]. This may be related to the possibility that indicators used in this study to measure the health of children are not sufficiently specific and detailed and may have limitations [38].

Our research still has some limitations. (1) We used self-rated health as one of the dependent variables. Self-rated health is a broad and biased variable. Moreover, there are differences in people’s understanding of the concept of ‘health.’ Some people may rate themselves as “sick” or in poor health due to diseases unrelated to their health (such as mental illness, cancer). (2) The explanatory variable of this study was the prevalence rate of sanitary toilets. Our standards for defining sanitary toilets were not comprehensive enough; as long as toilets could be flushed indoors, they met the standards for sanitary toilets. In fact, having only indoor flushing toilets is not a good indicator. Houses can be equipped with toilets, but without sewage treatment and hand washing facilities, these toilets may not meet the true standards of sanitary toilets. (3) Due to limitations of the data used, we were unable to observe whether the relevant facilities were equipped. (4) Although our study used longitudinal survey data, we only collected data at two-time points (2012 and 2014), and the tracking time was too short to observe long-term changes in health.

## Conclusion

Based on CFPS data from 2012 to 2014, we used the TW-FE model to test the impact of the popularity of village sanitary toilets on the health of rural residents. The results showed the following: (1) A significant positive relationship exists between the popularization of sanitary toilets and the health of rural residents. (2) The popularization of sanitary toilets is not only beneficial to the health of family members who use sanitary toilets but also to the health of those who do not use them. Their popularity has a significantly positive externality. (3) The popularity of sanitary toilets has a greater impact on children’s health, and their use is conducive to reducing the probability and frequency of children’s illness. (4) The popularity of sanitary toilets has significant regional differences in the health of rural residents, but gender differences are not significant.

To prevent and reduce the occurrence and spread of diseases and reduce poverty caused by diseases, we should continuously improve the construction level of rural settlements, pay attention to the externalities brought about by the improvement of the sanitary conditions of rural toilets, and emphasize the health protection effect of sanitary toilets. The popularity of sanitary toilets is of great importance to the health of rural residents, particularly children. To effectively promote the use of sanitary toilets in developing countries, improving the health of farmers’ children should be the initial focus. Additionally, raising awareness about the importance of using sanitary toilets and fostering enthusiasm among rural residents to adopt this practice are important approaches.

### Electronic supplementary material

Below is the link to the electronic supplementary material.

Supplementary Material 1

## Data Availability

This study analyzed publicly available datasets, available at: http://www.isss.pku.edu.cn/cfps/download.

## Footnotes

1 As long as the indoor flushing conditions are met, it can be recognized as a sanitary toilet.

## Abbreviations

CFPS: China Family Panel Studies
TW-FE: two-way fixed effect
