# Supplementary material for: Health effects and externalities of the popularization of sanitary toilets: evidence from Rural China
Source: BMC Public Health. 2023 Nov 11;23:2225. doi: 10.1186/s12889-023-17192-4 (PMC10640730; doi:10.1186/s12889-023-17192-4)
Supplement: Supplementary file 1 — Supplementary Material 1 [file 12889_2023_17192_MOESM1_ESM.docx]

**Table 4. Impact of sanitary toilets’ popularization rate on the health of adult rural residents**

|  | (1) | (2) | (3) |
| --- | --- | --- | --- |
| Variable | Self-rated health | Discomfort in the past two weeks | Diarrhea in the past two weeks |
| Popularization rate of sanitary toilets | 0.020 | -0.090*** | -0.134*** |
|  | (0.021) | (0.028) | (0.027) |
| Age | 0.018*** | 0.012*** | 0.002 |
|  | (0.002) | (0.003) | (0.002) |
| Married | 0.011 | -0.029 | 0.019 |
|  | (0.019) | (0.025) | (0.025) |
| Primary school | 0.044*** | -0.049*** | -0.019 |
|  | (0.014) | (0.016) | (0.012) |
| Junior high school | 0.063*** | -0.074*** | -0.042** |
|  | (0.020) | (0.023) | (0.020) |
| High school | 0.052* | -0.113*** | 0.001 |
|  | (0.027) | (0.032) | (0.036) |
| University and above | 0.004 | -0.127** | 0.421*** |
|  | (0.040) | (0.055) | (0.080) |
| Number of family members | 0.002 | 0.003 | -0.002 |
|  | (0.003) | (0.004) | (0.003) |
| Household per capita income in last year (natural logarithm) | 0.005 | 0.004 | -0.002 |
|  | (0.003) | (0.003) | (0.003) |
| Household per capita income in last year (natural logarithm) | 0.003 | 0.005** | 0.001 |
|  | (0.002) | (0.002) | (0.002) |
| Tap water and purified water | -0.001 | 0.001 | -0.002 |
|  | (0.009) | (0.010) | (0.009) |
| Availability of public garbage cans in village | 0.015* | -0.009 | 0.004 |
|  | (0.009) | (0.010) | (0.009) |
| _cons | -0.212** | -0.237** | -0.066 |
|  | (0.101) | (0.120) | (0.122) |
| Obs. | 39671 | 35499 | 11200 |
| R-squared | 0.127 | 0.123 | 0.116 |

Note: The table presents the coefficients from estimation of the TW-FE model with robust standard errors in parentheses. *** p≤0.01, ** p≤0.05, * p≤0.1.

**Table 5. Impact of sanitary toilets’ popularization rate on the health of adult rural residents**

|  | (4) | (5) | (6) | (7) | (8) | (9) |
| --- | --- | --- | --- | --- | --- | --- |
| Variable | Self-rated health | Discomfort in the past two weeks | Diarrhea in the past two weeks | Self-rated health | Discomfort in the past two weeks | Diarrhea in the past two weeks |
| Popularization rate of sanitary toilets | 0.074 | -0.129** | -0.165*** | 0.082** | -0.085* | -0.132*** |
|  | (0.047) | (0.064) | (0.055) | (0.035) | (0.048) | (0.044) |
| Age | 0.017*** | -0.004 | 0.006 | 0.019*** | 0.016*** | 0.002 |
|  | (0.006) | (0.007) | (0.006) | (0.003) | (0.003) | (0.003) |
| Married | 0.088* | -0.004 | 0.020 | -0.006 | -0.064** | 0.024 |
|  | (0.047) | (0.058) | (0.057) | (0.023) | (0.031) | (0.029) |
| Primary school | 0.006 | -0.020 | -0.049* | 0.042** | -0.054*** | -0.019 |
|  | (0.032) | (0.035) | (0.027) | (0.017) | (0.019) | (0.014) |
| Junior high school | 0.052 | -0.076 | -0.105** | 0.054** | -0.062** | -0.047** |
|  | (0.048) | (0.053) | (0.048) | (0.024) | (0.027) | (0.024) |
| High school | 0.023 | -0.058 | -0.018 | 0.040 | -0.123*** | -0.047 |
|  | (0.064) | (0.073) | (0.079) | (0.033) | (0.039) | (0.045) |
| University and above | 0.039 | 0.005 | -0.039 | -0.014 | -0.148** | 0.472*** |
|  | (0.092) | (0.119) | (0.205) | (0.048) | (0.069) | (0.103) |
| Number of family members | 0.003 | 0.005 | -0.007 | 0.007* | 0.005 | -0.000 |
|  | (0.007) | (0.008) | (0.006) | (0.004) | (0.005) | (0.004) |
| Household per capita income in last year (natural logarithm) | -0.006 | 0.008 | -0.002 | 0.003 | 0.004 | -0.002 |
|  | (0.007) | (0.008) | (0.006) | (0.003) | (0.004) | (0.003) |
| Household per capita income in last year (natural logarithm) | -0.005 | -0.003 | 0.001 | 0.003 | 0.007** | -0.003 |
|  | (0.005) | (0.005) | (0.004) | (0.002) | (0.003) | (0.002) |
| Tap water and purified water | -0.022 | 0.042 | 0.003 | 0.001 | -0.005 | -0.012 |
|  | (0.023) | (0.026) | (0.021) | (0.010) | (0.012) | (0.010) |
| Availability of public garbage cans in village | 0.024 | -0.024 | 0.004 | 0.006 | -0.015 | 0.006 |
|  | (0.022) | (0.025) | (0.021) | (0.011) | (0.013) | (0.012) |
| _cons | -0.199 | 0.436 | -0.235 | -0.308** | -0.399*** | -0.085 |
|  | (0.242) | (0.284) | (0.272) | (0.123) | (0.147) | (0.146) |
| Obs. | 10793 | 9788 | 2911 | 28878 | 25771 | 8289 |
| R-squared | 0.109 | 0.104 | 0.125 | 0.108 | 0.104 | 0.118 |

Note: The table presents coefficients from estimation of the TW-FE model with robust standard errors in parentheses. *** p≤0.01, ** p≤0.05, * p≤0.1.

**Table 6. Impact of sanitary toilet prevalence on rural children’s health**

|  | (1) | (2) | (3) | (4) | (5) | (6) |
| --- | --- | --- | --- | --- | --- | --- |
| Variable | Self-rated health | Were you sick in the past month? | Number of episodes of illnesses in the past month | Self-rated health | Were you sick in the past month? | Number of episodes of illnesses in the past month |
| Popularization rate of sanitary toilets | 0.499* | -0527** | -1.244*** | 0.179 | -0.673** | -2.360** |
|  | (0.296) | (0.263) | (0.235) | (0.276) | (0.291) | (1.155) |
| Age | 0.012 | -0.596*** | -1.447*** | 0.037 | -0.614*** | -1.468*** |
|  | (0.016) | (0.014) | (0.033) | (0.028) | (0.024) | (0.056) |
| Enrolled in school | 0.072** | 0.021 | 0.290*** | 0.082 | 0.052 | 0.155 |
|  | (0.030) | (0.026) | (0.062) | (0.057) | (0.048) | (0.112) |
| The number of family members | 0.026 | -0.009 | 0.078 | -0.070 | -0.082 | 0.135 |
|  | (0.052) | (0.045) | (0.109) | (0.093) | (0.076) | (0.179) |
| Household per capita income in last year (natural logarithm) | 0.092** | 0.042 | -0.122 | 0.169** | 0.034 | -0.156 |
|  | (0.043) | (0.038) | (0.091) | (0.078) | (0.066) | (0.156) |
| Congestion degree of family residence | 0.146*** | 0.069*** | -0.152** | 0.129** | 0.118*** | -0.243** |
|  | (0.029) | (0.025) | (0.061) | (0.053) | (0.045) | (0.106) |
| Tap water and purified water | 0.173 | -0.100 | 0.180 | 0.025 | -0.427* | 0.765 |
|  | (0.131) | (0.115) | (0.276) | (0.310) | (0.255) | (0.600) |
| Availability of public garbage cans in village | -0.106 | 0.100 | -0.323 | -0.294 | -0.311 | 0.364 |
|  | (0.135) | (0.118) | (0.286) | (0.267) | (0.220) | (0.519) |
| _cons | 3.408*** | -2.914*** | 10.467*** | 3.404*** | -2.894*** | 11.408*** |
|  | (0.497) | (0.437) | (1.052) | (0.937) | (0.793) | (1.866) |
| Obs. | 7363 | 7363 | 7363 | 3163 | 3136 | 3136 |
| R-squared | 0.234 | 0.604 | 0.604 | 0.238 | 0.633 | 0.635 |

Note: The table presents coefficients from estimation of the TW-FE model with robust standard errors in parentheses. *** p≤0.01, ** p≤0.05, * p≤0.1.

**Table 7. Impact of sanitary toilet prevalence on rural children’s health**

|  | (1) | (2) | (3) |
| --- | --- | --- | --- |
| Variable | Self-rated health | Were you sick in the past month? | Number of episodes of illnesses in the past month |
| Popularization rate of sanitary toilets | 0.465 | -0.526** | -1.940*** |
|  | (0.313) | (0.229) | (0.514) |
| Age | -0.004 | -0.577*** | -1.415*** |
|  | (0.022) | (0.019) | (0.046) |
| Enrolled in school | 0.072* | 0.002 | 0.374*** |
|  | (0.040) | (0.034) | (0.082) |
| The number of family members | 0.161* | -0.013 | 0.183 |
|  | (0.091) | (0.078) | (0.192) |
| Household per capita income in last year (natural logarithm) | 0.083 | 0.062 | -0.170 |
|  | (0.060) | (0.052) | (0.126) |
| Congestion degree of family residence | 0.155*** | 0.049 | -0.124 |
|  | (0.041) | (0.035) | (0.086) |
| Tap water and purified water | 0.293* | 0.106 | -0.249 |
|  | (0.167) | (0.143) | (0.349) |
| Availability of public garbage cans in village | -0.102 | 0.221 | -0.475 |
|  | (0.191) | (0.166) | (0.406) |
| _cons | 2.903*** | -2.755*** | 9.548*** |
|  | (0.742) | (0.637) | (1.557) |
| Obs. | 4200 | 4200 | 4200 |
| R-squared | 0.238 | 0.588 | 0.587 |

Note: The table presents coefficients from estimation of the TW-FE model with robust standard errors in parentheses. *** p≤0.01, ** p≤0.05, * p≤0.1.

**Table 8. Impact of the prevalence of sanitary toilets on adult rural residents’ health: by region**

|  | (1) | (2) | (3) | (4) | (5) | (6) | (7) | (8) | (9) |
| --- | --- | --- | --- | --- | --- | --- | --- | --- | --- |
|  | Eastern regions | | | Central regions | | | Western regions | | |
| Variable | Self-rated health | Discomfort in the past two weeks | Diarrhea in the past two weeks | Self-rated health | Discomfort in the past two weeks | Diarrhea in the past two weeks | Self-rated health | Discomfort in the past two weeks | Diarrhea in the past two weeks |
| Popularization rate of sanitary toilets | 0.086 | -0.203** | -0.131 | 0.005 | -0.193** | -0.155** | 0.157*** | 0.123 | -0.129** |
|  | (0.064) | (0.085) | (0.099) | (0.060) | (0.076) | (0.077) | (0.061) | (0.091) | (0.054) |
| Age | 0.020*** | 0.015** | 0.001 | 0.021*** | 0.014*** | 0.006 | 0.019*** | 0.018*** | 0.001 |
|  | (0.005) | (0.006) | (0.007) | (0.005) | (0.005) | (0.005) | (0.005) | (0.006) | (0.003) |
| Married | 0.046 | -0.093* | 0.188*** | 0.047 | -0.012 | -0.052 | -0.074** | -0.087* | -0.066 |
|  | (0.045) | (0.056) | (0.064) | (0.041) | (0.050) | (0.046) | (0.037) | (0.052) | (0.040) |
| Primary school | 0.036 | -0.059* | -0.025 | -0.000 | -0.027 | -0.016 | 0.071*** | -0.066** | -0.012 |
|  | (0.028) | (0.032) | (0.031) | (0.034) | (0.036) | (0.029) | (0.026) | (0.031) | (0.017) |
| Junior high school | 0.022 | -0.084* | -0.037 | 0.018 | -0.038 | -0.067 | 0.101** | -0.050 | -0.038 |
|  | (0.039) | (0.046) | (0.051) | (0.045) | (0.049) | (0.043) | (0.040) | (0.048) | (0.030) |
| High school | 0.040 | -0.098 | -0.153 | -0.011 | -0.049 | -0.079 | 0.078 | -0.187*** | 0.166** |
|  | (0.059) | (0.070) | (0.103) | (0.062) | (0.070) | (0.069) | (0.053) | (0.065) | (0.075) |
| University and above | -0.056 | -0.068 | 0.501*** | -0.073 | -0.140 | / | 0.059 | -0.202* | 0.654*** |
|  | (0.087) | (0.123) | (0.183) | (0.087) | (0.118) | / | (0.079) | (0.117) | (0.131) |
| Number of family members | -0.006 | 0.007 | 0.003 | 0.010 | 0.008 | 0.006 | 0.011 | -0.001 | -0.012** |
|  | (0.008) | (0.010) | (0.010) | (0.007) | (0.007) | (0.006) | (0.007) | (0.008) | (0.005) |
| Household per capita income in last year (natural logarithm) | 0.004 | 0.011 | -0.006 | 0.003 | -0.009 | 0.002 | 0.002 | 0.007 | -0.003 |
|  | (0.006) | (0.007) | (0.008) | (0.006) | (0.007) | (0.006) | (0.005) | (0.006) | (0.004) |
| Household per capita income in last year (natural logarithm) | 0.001 | -0.001 | 0.001 | 0.008* | 0.006 | 0.001 | 0.001 | 0.011** | -0.002 |
|  | (0.004) | (0.005) | (0.006) | (0.004) | (0.005) | (0.004) | (0.004) | (0.005) | (0.003) |
| Tap water and purified water | 0.002 | -0.011 | -0.036 | -0.011 | 0.001 | -0.026 | 0.003 | -0.009 | 0.007 |
|  | (0.019) | (0.022) | (0.026) | (0.018) | (0.021) | (0.017) | (0.017) | (0.020) | (0.012) |
| Availability of public garbage cans in village | -0.009 | -0.003 | -0.005 | -0.005 | 0.010 | 0.016 | 0.036* | -0.044* | 0.010 |
|  | (0.017) | (0.020) | (0.024) | (0.022) | (0.024) | (0.020) | (0.020) | (0.024) | (0.015) |
| _cons | -0.323 | -0.343 | -0.129 | -0.451** | -0.303 | -0.260 | -0.241 | -0.453* | 0.098 |
|  | (0.222) | (0.265) | (0.367) | (0.220) | (0.252) | (0.246) | (0.201) | (0.249) | (0.173) |
| Obs. | 9082 | 8488 | 4630 | 19717 | 21935 | 3430 | 10782 | 9248 | 3140 |
| R-squared | 0.119 | 0.116 | 0.139 | 0.110 | 0.114 | 0.118 | 0.112 | 0.119 | 0.160 |

Note: The table presents coefficients from estimation of the TW-FE model with robust standard errors in parentheses. *** p≤0.01, ** p≤0.05, * p≤0.1.

**Table 9. Impact of sanitary toilet prevalence on children’s health: by region**

|  | (1) | (2) | (3) | (4) | (5) | (6) | (7) | (8) | (9) |
| --- | --- | --- | --- | --- | --- | --- | --- | --- | --- |
|  | Eastern regions | | | Central regions | | | Western regions | | |
| Variable | Self-rated health | Were you sick in the past month? | Number of times ill in the past month | Self-rated health | Were you sick in the past month? | Number of times ill in the past month | Self-rated health | Were you sick in the past month? | Number of times ill in the past month |
| Popularization rate of sanitary toilets | 0.314 | -0.819* | -0.031 | 2.486*** | -0.976** | 3.459*** | 0.380 | -0.859** | -2.289** |
|  | (1.275) | (0.479) | (1.067) | (0.884) | (0.405) | (1.260) | (1.116) | (0.425) | (1.113) |
| Age | 0.070 | 0.498*** | -1.194*** | 0.012 | -0.589*** | -1.409*** | 0.056* | -0.616*** | -1.536*** |
|  | (0.056) | (0.039) | (0.091) | (0.031) | (0.031) | (0.077) | (0.034) | (0.031) | (0.075) |
| Enrolled in school | 0.199* | -0.024 | 0.194 | 0.050 | 0.055 | 0.350** | 0.042 | -0.012 | 0.421*** |
|  | (0.119) | (0.081) | (0.190) | (0.060) | (0.060) | (0.150) | (0.054) | (0.049) | (0.119) |
| The number of family members | 0.482 | -0.024 | 0.050 | 0.080 | -0.151 | 0.550* | 0.058 | 0.056 | -0.154 |
|  | (0.317) | (0.218) | (0.512) | (0.114) | (0.117) | (0.291) | (0.145) | (0.134) | (0.328) |
| Household per capita income in last year (natural logarithm) | 0.003 | 0.306** | -0.692** | 0.089 | 0.007 | -0.030 | 0.099 | 0.008 | -0.054 |
|  | (0.186) | (0.126) | (0.297) | (0.085) | (0.087) | (0.216) | (0.082) | (0.076) | (0.186) |
| Congestion degree of family residence | 0.287** | 0.021 | -0.062 | 0.198*** | 0.038 | -0.113 | 0.044 | 0.060 | -0.167 |
|  | (0.124) | (0.086) | (0.201) | (0.053) | (0.053) | (0.132) | (0.065) | (0.059) | (0.144) |
| Tap water and purified water | 0.118 | 0.389 | -0.848 | 0.225 | 0.102 | -0.197 | 0.389 | -0.038 | 0.135 |
|  | (0.433) | (0.301) | (0.706) | (0.254) | (0.260) | (0.648) | (0.237) | (0.213) | (0.522) |
| Availability of public garbage cans in village | -0.096 | 0.380 | -0.819 | 0.022 | 0.283 | -0.609 | -0.122 | 0.250 | -0.592 |
|  | (0.393) | (0.273) | (0.642) | (0.320) | (0.325) | (0.811) | (0.315) | (0.295) | (0.723) |
| _cons | 2.127 | -4.331*** | 13.727*** | 3.138*** | -1.425 | 5.626** | 3.119*** | -3.083*** | 11.716*** |
|  | (2.221) | (1.538) | (3.615) | (1.019) | (1.032) | (2.577) | (1.115) | (1.023) | (2.508) |
| Obs. | 3132 | 2853 | 3151 | 2436 | 2597 | 2562 | 1795 | 1913 | 1650 |
| R-squared | 0.149 | 0.563 | 0.564 | 0.100 | 0.602 | 0.594 | 0.043 | 0.612 | 0.615 |

Note: The table presents coefficients from estimation of the TW-FE model with robust standard errors in parentheses. *** p≤0.01, ** p≤0.05, * p≤0.1.

**Table 10. Impact of the prevalence of sanitary toilets on adult rural residents’ health: by gender**

|  | (1) | (2) | (3) | （4） | （5） | （6） |
| --- | --- | --- | --- | --- | --- | --- |
|  | Adult males | | | Adult females | | |
| Variable | Self-rated health | Discomfort in the past two weeks | Diarrhea in the past two weeks | Self-rated health | Discomfort in the past two weeks | Diarrhea in the past two weeks |
| Popularization rate of sanitary toilets | 0.094** | -0.119 | -0.147** | -0.067 | -0.158** | -0.231*** |
|  | (0.047) | (0.066) | (0.073) | (0.053) | (0.070) | (0.054) |
| Age | 0.014*** | 0.010** | -0.001 | 0.026*** | 0.021*** | 0.004 |
|  | (0.004) | (0.005) | (0.005) | (0.004) | (0.005) | (0.003) |
| Married | -0.040 | -0.033 | 0.071 | 0.050 | -0.090** | 0.006 |
|  | (0.030) | (0.042) | (0.057) | (0.036) | (0.044) | (0.034) |
| Primary school | 0.036 | -0.017 | -0.017 | 0.049** | -0.088*** | -0.018 |
|  | (0.023) | (0.026) | (0.024) | (0.025) | (0.028) | (0.018) |
| Junior high school | 0.051 | -0.020 | -0.042 | 0.056 | -0.099** | -0.047 |
|  | (0.032) | (0.036) | (0.038) | (0.036) | (0.041) | (0.031) |
| High school | 0.024 | -0.082 | -0.030 | 0.057 | -0.159*** | -0.050 |
|  | (0.045) | (0.053) | (0.082) | (0.048) | (0.058) | (0.055) |
| University and above | 0.003 | -0.021 | \ | -0.039 | -0.269*** | 0.468*** |
|  | (0.066) | (0.093) | \ | (0.070) | (0.101) | (0.101) |
| Number of family members | 0.000 | 0.001 | 0.001 | 0.016*** | 0.008 | -0.000 |
|  | (0.006) | (0.006) | (0.007) | (0.006) | (0.007) | (0.005) |
| Household per capita income in last year (natural logarithm) | 0.006 | 0.002 | 0.004 | -0.001 | 0.005 | -0.006 |
|  | (0.005) | (0.005) | (0.006) | (0.005) | (0.006) | (0.004) |
| Household per capita income in last year (natural logarithm) | 0.002 | 0.006* | -0.000 | 0.004 | 0.007* | -0.001 |
|  | (0.003) | (0.004) | (0.004) | (0.004) | (0.004) | (0.003) |
| Tap water and purified water | 0.019 | 0.010 | -0.026 | -0.016 | -0.021 | -0.002 |
|  | (0.014) | (0.017) | (0.017) | (0.015) | (0.018) | (0.013) |
| Availability of public garbage cans in village | 0.037** | -0.019 | -0.012 | -0.028* | -0.012 | 0.018 |
|  | (0.015) | (0.018) | (0.019) | (0.016) | (0.018) | (0.014) |
| _cons | 0.023 | -0.218 | -0.011 | -0.690*** | -0.560*** | -0.120 |
|  | (0.170) | (0.205) | (0.260) | (0.178) | (0.210) | (0.175) |
| Obs. | 20434 | 19734 | 5764 | 19237 | 19937 | 5436 |
| R-squared | 0.108 | 0.103 | 0.115 | 0.113 | 0.108 | 0.130 |

Note: The table presents coefficients from estimation of the TW-FE model with robust standard errors in parentheses. *** p≤0.01, ** p≤0.05, * p≤0.1.

**Table 11. Impact of sanitary toilet prevalence on children’s health: by gender**

|  | (1) | (2) | (3) | （4） | （5） | （6） |
| --- | --- | --- | --- | --- | --- | --- |
|  | Boys | | | Girls | | |
| Variable | Self-rated health | Were you sick in the past month? | Number of times ill in the past month | Self-rated health | Were you sick in the past month? | Number of times ill in the past month |
| Popularization rate of sanitary toilets | -2.688 | 0.111 | -0.066 | 1.252** | -0.021 | -0.403 |
|  | (2.985) | (1.740) | (4.359) | (0.534) | (0.811) | (0.762) |
| Age | 0.114 | 0.647*** | -1.495*** | 0.040** | 0.582*** | -0.011 |
|  | (0.158) | (0.084) | (0.210) | (0.018) | (0.027) | (0.025) |
| Enrolled in school | 0.544** | -0.131 | 1.095*** | 0.012 | -0.027 | 0.186*** |
|  | (0.259) | (0.116) | (0.291) | (0.032) | (0.049) | (0.057) |
| The number of family members | 1.112 | 0.748 | -1.415 | 0.152** | 0.132 | 0.120 |
|  | (0.778) | (0.455) | (1.139) | (0.075) | (0.115) | (0.126) |
| Household per capita income in last year (natural logarithm) | 0.697 | 0.152 | -0.221 | 0.027 | 0.046 | -0.004 |
|  | (0.427) | (0.225) | (0.564) | (0.050) | (0.076) | (0.080) |
| Congestion degree of family residence | 0.120 | 0.062 | -0.063 | 0.101*** | 0.008 | -0.022 |
|  | (0.229) | (0.129) | (0.324) | (0.032) | (0.049) | (0.047) |
| Tap water and purified water | 1.850* | 0.421 | -0.907 | 0.414*** | 0.100 | 0.067 |
|  | (1.065) | (0.587) | (1.471) | (0.132) | (0.200) | (0.205) |
| Availability of public garbage cans in village | -1.806 | 0.903 | -2.282 | -0.161 | 0.510** | -0.012 |
|  | (1.215) | (0.707) | (1.770) | (0.163) | (0.251) | (0.225) |
| _cons | -9.094 | -8.711** | 19.340** | 3.371*** | -3.259*** | 0.833 |
|  | (6.219) | (3.442) | (8.620) | (0.592) | (0.906) | (0.993) |
| Obs. | 3839 | 3806 | 3806 | 3524 | 3637 | 3627 |
| R-squared | 0.232 | 0.716 | 0.725 | 0.119 | 0.596 | 0.182 |

Note: The table presents coefficients from estimation of the TW-FE model with robust standard errors in parentheses. *** p≤0.01, ** p≤0.05, * p≤0.1.
